# Recombinant Human Lactoferrin Reduces Inflammation and Increases Fluoroquinolone Penetration to Primary Granulomas during Mycobacterial Infection of C57Bl/6 Mice

**DOI:** 10.1007/s00005-022-00648-7

**Published:** 2022-02-28

**Authors:** Thao K.T. Nguyen, Zainab Niaz, Marian L. Kruzel, Jeffrey K. Actor

**Affiliations:** 1 Department of Pathology and Laboratory Medicine, UTHealth McGovern Medical School, Houston, TX, USA; 2 The University of Texas MD Anderson Cancer Center – UTHealth Graduate School of Biomedical Sciences, Houston, TX 77030, USA

**Keywords:** Lactoferrin, Mycobacterium tuberculosis, *Mtb*, Granuloma, Immunopathology, M1/M2 Phenotype, Tuberculosis

## Abstract

Infection with *Mycobacterium tuberculosis* (*Mtb*) results in the primary formation of a densely packed inflammatory foci that limits entry of therapeutic agents into pulmonary sites where organisms reside. No current therapeutic regimens exist that modulate host immune responses to permit increased drug penetration to regions of pathological damage during tuberculosis disease. Lactoferrin is a natural iron-binding protein previously demonstrated to modulate inflammation and granuloma cohesiveness, while maintaining control of pathogenic burden. Studies were designed to examine recombinant human lactoferrin (rHLF) to modulate histological progression of *Mtb*-induced pathology in a non-necrotic model using C57Bl/6 mice. The rHLF was oral administered at times corresponding to initiation of primary granulomatous response, or during granuloma maintenance. Treatment with rHLF demonstrated significant reduction in size of primary inflammatory foci following *Mtb* challenge, and permitted penetration of ofloxacin fluoroquinolone therapeutic to sites of pathological disruption where activated (foamy) macrophages reside. Increased drug penetration was accompanied by retention of endothelial cell integrity. Immunohistochemistry revealed altered patterns of M1-like and M2-like phenotypic cell localization post infectious challenge, with increased presence of M2-like markers found evenly distributed throughout regions of pulmonary inflammatory foci in rHLF treated mice.

## INTRODUCTION

Despite world-wide extensive research and eradicating efforts, *Mycobacterium tuberculosis* (*Mtb*) remains a major infectious pathogen to the human population with approximately 10.0 million infected cases and 1.5 million deaths reported in 2020 globally (World Health Organization 2020). The initial host-pathogen interaction and complex immunological responses culminate in an inflammatory pathology within pulmonary tissue, characterized as a primary granulomatous disease (Adams 1976; Hunter et al. 2006a). These granulomas and associated lesions obstruct normal pulmonary functions. Active research investigates how *Mtb* directly induces the granulomatous response, focusing on areas of pathological damage and bacterial burden that are most severe during primary infection.

*Mtb* associated factors are involved in the recognition and activation of host cells within the bronchial regions, leading to uptake by alveolar macrophages (Akira et al. 2006). This interaction triggers a series of immune responses via the production and release of cytokines by infected and responding cells (Hossain and Norazmi 2013). For example, trehalose 6’6-dimycolate (TDM), an abundant mycobacterial mycolic acid, directly triggers a granulomatous response (Hunter et al. 2006a; Nguyen et al. 2020) that resembles early (primary) infection pathology. TDM-activated macrophages release pro-inflammatory mediators, such as TNF-*α* and IL-1-β, along with additional chemotactic factors, to further recruit immune cells to areas of infection (Khader et al. 2009; Monin and Khader 2014; Welsh et al. 2008). Over time, additional recruited immune cells participate in formation of organized, sphere-shaped primary inflammatory structures (Martin et al. 2016). There exists a balance between host and organism; the granulomas formed during active *Mtb* infection contain and limit bacterial dissemination (Russell 2007), yet organisms have adopted mechanisms to survive and grow inside macrophage host cells. Indeed, recruited naïve macrophages can become new potential sites for *Mtb* to shelter and replicate (Davis and Ramakrishnan 2009).

Clinically, the physical nature of the primary granuloma also limits penetration of antimycobacterial therapeutics; as granulomas mature, reduction in vascularization limits drug delivery to within granulomas where large populations of *Mtb* may reside (Dartois 2014; Driver et al. 2012; Paige and Bishai 2010). This is not dissimilar to constraints seen in drug delivery to solid tumors (Minchinton and Tannock 2006). The physical nature of the host immune response therefore represents a challenge in treating *Mtb*-infected patients – it contributes to prolongation of treatment while permitting a small population of *Mtb* to escape elimination. The lack of complete penetration of antimycobacterial agents also indirectly increases risk of developing antibiotic-resistance (Kaplan et al. 2003, Schito et al. 2015, Sotgiu et al. 2015). Therefore, multiple lines of investigation currently are aimed at improving drug delivery through focused targeting that either manipulates the cohesiveness of the granuloma structure, or alters immune responses to *Mtb* during granuloma development (Kaplan 2020). These studies typically use models to mimic innate (primary) infection, or models with development of lesions with liquefactive necrosis (Driver et al. 2012).

Novel approaches to *Mtb* treatment include host-directed therapies (Ndlovu and Marakalala 2016) which are focused in two major fronts; the first augments immune response using immune-based treatments (Vilaplana et al. 2013), while the second alters the resultant immunopathology (Nguyen et al. 2021). Examples of immune-based treatment methods include agents that target the macro-autophagic compartment, such as vitamin D and retinoic acid, which increase phagocytosis to enhance the lysosomal degradative processes inside macrophages (Estrella et al. 2011). Ibuprofen, an anti-inflammatory drug, functions as an adjunct treatment by facilitating pyrazinamide to alleviate pathological damage in the lungs (Byrne et al. 2007; Eisen et al. 2013; Vilaplana et al. 2013). These immune-based approaches have significant potential, demonstrating superior disease outcomes in clinical trial results (Hayford et al. 2020; Zhang et al. 2018). While promising, the obstacle in drug delivery to macrophages inside established granulomas persists (Gupta et al. 2016). Therefore, therapies that target pathologies, in addition to antimycobacterial function, may subsequently be more useful as clinical tools. A well-known example of the immunopathological alteration approach is blocking excess TNF-α, a key pro-inflammatory cytokine required for granuloma formation (Flynn et al. 1995; Plessner et al. 2007). TNF-α inhibitors have been used to treat other inflammatory diseases effectively (Lv et al. 2014; Skerry et al. 2012; Zhang et al. 2021). However, its current downside is that TNF-α inhibitors can lead to increased bacterial dissemination; abolishing the granuloma containment modality runs the risk of *Mtb* reactivation (Chakravarty et al. 2008; Fernandez-Ruiz and Aguado 2018; Keane et al. 2001; Wallis et al. 2004).

Lactoferrin, a glycoprotein known for its ability to bind iron, has been extensively studied for its role as an immune modulator in host defense in disease models (Kruzel et al. 2017; Rosa et al. 2017). Lactoferrin falls into the category of “immune modulators”, and has been identified to reduce pathology of the tuberculoid granuloma (Hwang et al. 2017). As a modulator, lactoferrin demonstrates efficacy to boost immune memory responses in vaccine models, while reducing pro-inflammatory response in lipopolysaccharide exposed mouse models (Crouch et al. 1992; Hwang et al. 2009a, 2011; Kruzel et al. 2002; Legrand 2012). Relative to the study described in this report, bovine lactoferrin significantlly reduced inflammatory pathology in *Mtb*-infected mice (Welsh et al. 2011). Both human and bovine lactoferrins were also shown effective to limit inflammation in a non-infectious TDM-induced granuloma mouse model (Nguyen et al. 2021; Welsh et al. 2010).

Lactoferrin treatment reduced the M1 phenotypic response to TDM, and limits pathological damage in murine lungs (Nguyen et al. 2020, 2021). The lactoferrin treatment also significantly increased penetration of a second-line anti-mycobacterial agent fluoroquinolone into TDM-induced granulomas (Nguyen et al. 2021). Such anti-inflammatory response in the lactoferrin-treated mice, along with other data showing lactoferrin reduces pro-inflammatory phenotypes in macrophages (Hwang et al. 2016; Wisgrill et al. 2018), suggests that lactoferrin had a modulating pro-inflammatory response on granulomas. The studies presented here extend these observations to examine if lactoferrin can modulate primary granuloma permeability and drug distribution during active *Mtb* infection. Here, the effect of lactoferrin on the permeability of primary granulomas (non-necrotic) to a fluoroquinolone is examined in the *Mtb*-infected C57Bl/6 mouse model, when lactoferrin is administered as a prophylactic (defined as when given prior to granuloma formation) or therapeutic (defined as when given after granuloma establishment) intervention. These experiments shed light on mechanisms underlying changes to the early *Mtb* pathological events due to lactoferrin adjunct treatment.

## MATERIALS AND METHODS

### Mice

Five-week-old female C57BL/6 mice were purchased from Envigo (Houston, TX) with 18–20 g initial body mass. Eight to ten mice were used per group, per time each point indicated. All *Mtb* infections occurred in biosafety level 3 facilities under the University of Texas Health Science Center at Houston institutional guidelines (IBC-18-014), with approval from the animal ethics committee (AWC-17-0089).

### Recombinant Human Lactoferrin and Ofloxacin, and Delivery to Mice

CHO-expressed recombinant human lactoferrin (rHLF; >98% purity; <10% iron saturated; <0.5 EU·mg^−1^; Cat# LFH-101) was kindly provided as lyophilized powder by PharmaReview Corporation (Houston, TX) (Choi et al. 2008; Kruzel et al. 2021). The rHLF was reconstituted in dH_2_O to a concentration of 10 mg·mL^−1^. From day 14 to day 28 after *Mtb* infection, mice were given 1 mg·(100 μL)^−1^·mouse^−1^ of rHLF by oral gavage every other day as prophylatic treatment, similar to reported use in models of mycobacterial granulomatous responses (Hwang et al. 2017; Welsh et al. 2010, 2011). One mg/ml was found to be more productive than a 100 μg/ml dose (Fig. S.1). From day 21 to day 28 after *Mtb* infection, another group of mice were given 1 mg·(100 μL)^−1^·mouse^−1^ of recombinant human lactoferrin by oral gavage every other day as therapeutic treatment. Ofloxacin (Sigma Life Science; O8757-1G) was given at 100 μL of 30 mg·mL^·1^·mouse^−1^, solubilized in DMSO and diluted 1:10 with phosphate buffered saline (PBS), was intraperitoneal administered 30 min prior to sacrifice (Batard et al. 2011; Hwang et al. 2008).

### Mtb Infection

**Fig. 1.**
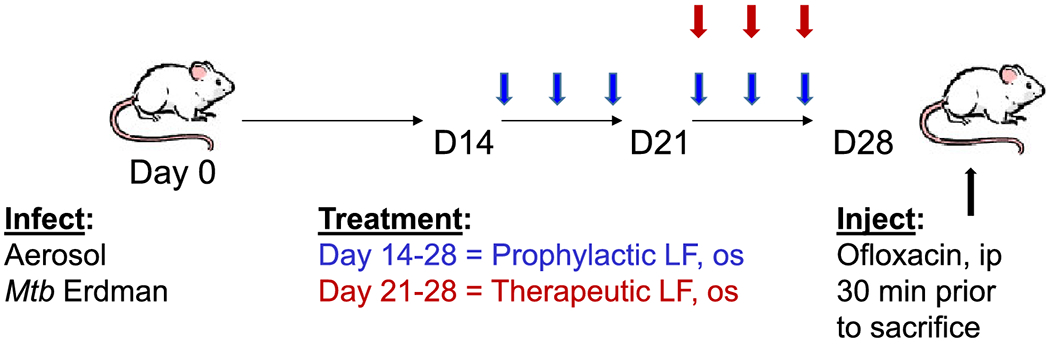
*Mtb* infection and lactoferrin treatment scheme. C57Bl/6 mice were aerosol challenged with *Mtb* and treated with rHLF administered every other day orally beginning on day 14 (prophylactic relative to granulomatous responses; six total doses), or beginning on day 21 post infection (therapeutic relative to established granulomas; three total doses). Mice were intravenous injected with ofloxacin 30 min prior to sacrifice, at 28 days post initial infectious challenge.

Aerosol infections were done as previously reported (Hwang et al. 2009c, 2011), using *Mycobacterium tuberculosis*, strain Erdman (TMC 107, American Type Cell Culture). Organisms were cultured in Middlebrook 7H9 broth with 10% supplement (5% bovine serum albumin, 2% dextrose, and 0.5% Tween 20 in distilled water) to log phase. Pelleted bacteria were resuspended in PBS and diluted to 3 × 10^8^ colony forming units (CFU) per ml using McFarland standards. Bacteria were sonicated to disperse aggregates. The bacterial CFUs for each time point, including day 1 post infection, were confirmed by plating serial dilutions on Middlebrook 7H11 agar plates (Hardy Diagnostics, Santa Maria, CA) using the large right lobe of the mouse lung that was weighed and homogenized into 2 mL PBS, which were incubated at 37°C for 3 – 4 weeks. Mice were infected for four weeks, with treatments described above, using the protocol shown in Fig. 1. Bovine lactoferrin, shown to have biological equivalency to the rHLF (Hwang et al. 2017) was used for analysis of CFU dissemination to other organs and to reconfirm bioequivalency in dose response treatments (Fig. S.2).

### Histological Assessment

The small left lobe of the mouse lung was collected and fixed in 10% buffered formalin. For histologic analysis, the lung was sectioned (5 μm thick) and stained with hematoxylin and eosin (H&E) and acid-fast staining as per standard procedures (Hwang et al. 2011). The histological assessment of the lung tissue following aerosol infection was done as previously reported (Hwang et al. 2009c). Multiple sections from at least 6 mice per group were analyzed using Motic DSAssistant digital software (version 1.0.7.44; Kowloon Bay, Kowloon, HK) (Nguyen et al. 2021). H&E stained and acid-fast stained slides were viewed by a trained pathologist, with descriptive results obtained in an experimentally blinded manner.

### Immunohistochemistry

Fixed lung was embedded in paraffin, sectioned, and stained for immunohistochemical examination, similar to methods described (Hwang et al. 2019), diluted at 1:2000, was performed according to manufacturer’s instructions with a modification of 20 min at low pH for antigen retrival, and visualized using standard HRP techniques and DAB chromogen using Dako reagents (Dako, Agilent, Santa Clara, CA). In a similar manner, M1-like marker CD38 (Invitrogen, ThermoFisher, Cat# 14-0381-02), diluted at 1:1000, M2-like marker CD 206 (Bioss, Cat# bs-4727R), diluted at 1:1000, and endothelial cell marker CD31/PECAM-1 (Cell Signaling, Cat# 77699T) diluted at 1:200 were used for visualization on serial slide sections. Hematoxylin counterstained slides were viewed by a trained pathologist, with descriptive results obtained in an experimentally blinded manner.

### Quantitative Assessment of Pulmonary Inflammation

High resolution scanned images of H&E-stained slides were assessed for lung inflammation and granulomas using Motic DSAssistant software (Kowloon Bay, Kowloon, HK), in a two step process using Fiji ImageJ (version 1.52o 23 April 2019, National Institutes of Health, Bethesda, MD) with plugin MorphoLibJ (McQuin et al. 2018), described in part in (Nguyen et al. 2021). Minimum and maximum values for hue, saturation, and brightness were set at: 120, 255; 0, 255; and 0, 255, respectively. Total cell area measurement used a modified equation detailed elsewhere where peak threshold was set at 164 (Schneider et al. 2012) (Fig. S.3). Values were averaged within treatment groups and normalized to non-treated controls.

H&E-stained slides were also used to capture photos of granulomas under the Olympus BX51 microscope using the Nuance Cri Multispectral Imaging System FX (PerkinElmer). The granuloma section was first identified and captured under H&E brightfield scope, then ofloxacin’s fluorescent signals were captured under 40× lens with FITC filter (emission restriction set between 540–560 nm) after 120 ms of exposure. All microscopic settings and factors were maintained throughout the photo taking process with image files having the same dimensions of 1392 × 1040 with 72 dpi resolution. For each granuloma, the total fluorescent area or ofloxacin absorption area and the total granuloma area were measured in pixel units using CellProfiler (software version 3.1.5) pipeline algorithm (McQuin et al. 2018), with described modifications (Diem et al. 2015). The measurement on the selected lung section is reported as percent area that represents the ratio of ofloxacin absorption over the total granuloma area, based on original observations of (Batard et al. 2011). All data was graphed and statistical analyzed in GraphPad Prism (version 5.03).

### Statistical Analysis

Data obtained was compared across groups then analyzed using a paired Student *t* test or one-way ANOVA with a Tukey post-hoc test; differences between means were considered statistically significant at a value of *p* ≤ 0.05. Data are presented are a combination of 2–3 experimental repeats. Each experiment incorporated 8–10 mice per group.

## RESULTS

### Oral Administration of Recombinant Human Lactoferrin Alleviates Pulmonary Inflammation Post Mtb Infection

**Fig. 2.**
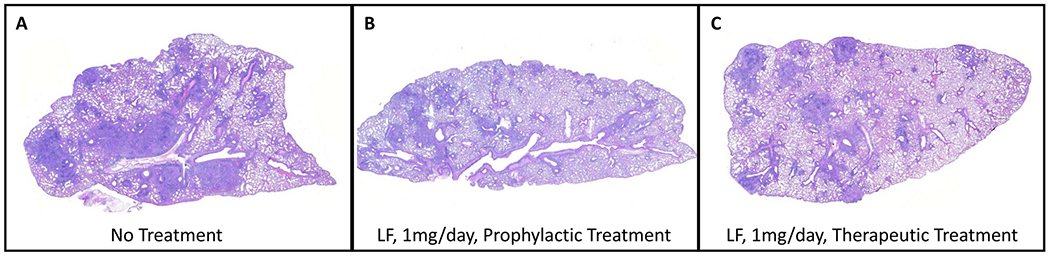
Lactoferrin treatment reduces pulmonary inflammation post infectious challenge with *Mtb*. Lungs from *Mtb* infected mice were assessed at day 28 post aerosol infection (A) and compared to animals given rHLF in the prophylactic group (B) or therapeutic group (C). Histologic assessment revealed primary granulomatous response with monocytic cell infiltration, dense cellular foci, and occluded vascular regions in control infected mice (4× magnification). Both prophylactic and therapeutic rHLF treatment reduced inflammatory response resulting in modest inflammatory foci and reduced pathological damage to lung tissue. Hematoxylin and eosin stained histographs represent formalin fixed lung sections at 10× magnification obtained from repeated studies with 8–10 mice in each group; study representative of repeat experiments.

Previous studies using bovine derived lactoferrin given in drinking water demonstrated that continuous-access oral delivery during *Mycobacterium tuberculosis* infection could reduce inflammation-related primary granulomatous pathology in mice (Welsh et al. 2011). A defined protocol was adopted to determine if Rhlf would initiate a similar protective response (Fig. 1). Mice were aerosol infected with *Mtb*, strain Erdman, and rHLF was given by oral gavage beginning either prior to (prophylactic), or post (therapeutic) expected initiation of granulomatous responses. Histological assessment (Fig. 2 and Fig. S.1) revealed marked reduction of pulmonary inflammation in both the prophylactic and therapeutic rHLF treated groups, in a similar manner to that previously reported with the bovine LF treatment. Specifically, the rHLF treatments demonstrated reduced complexity of granulomatous responses, smaller foci of inflammation, with less cellular accumulation and density in areas of inflammation. Quantitative measurement of inflammation confirmed histological reduction in pathology due to the rHLF treatments.

**Fig. 3.**
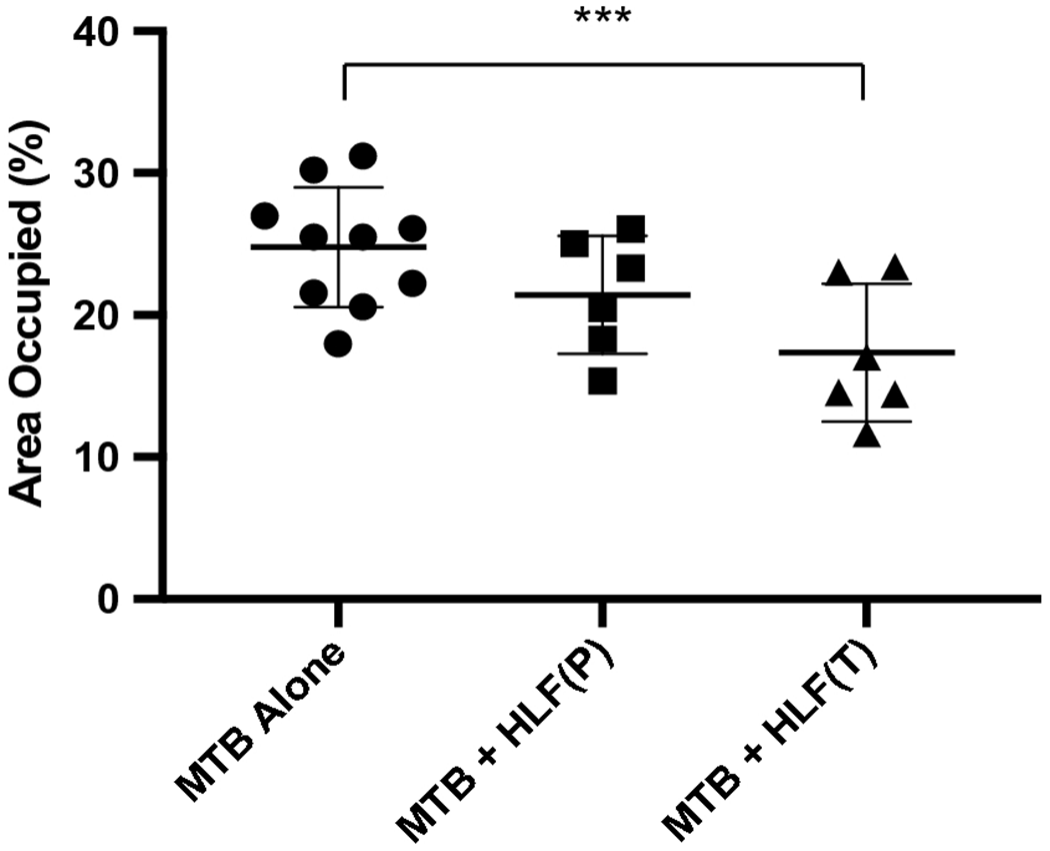
Decreased inflammatory response in lactoferrin treated mice. *Mtb* infected mice were assessed by digital analysis for cellularity and inflammation. Area occupied of pulmonary scanned sections is shown for individual mice, with and without rHLF treatments. Results represent mean ± standard deviation of the mean. Similar data was obtained in repeated experiments; 6–10 mice were included per group, per experiment. P: prophylactic treatment; T: therapeutic treatment; ***p≤0.001, one way ANOVA, Tukey post hoc test for multiple comparisons.

Whole right lobes of mouse lung were collected at four weeks post infections and processed for quantitative assessment of primary granulomatous response. Serial sections of H&E stained tissue sections were high resolution scanned to assess area occupied by inflammation (Fig. 3). The *Mtb* infected group had the largest occluded regions, with 24.79% ± 4.2% relative area occupied by inflammatory response. Both lactoferrin treatment modalities resulted in reduction of pathology. The prophylactic treatment resulted in reduction of granuloma area to 21.42% ± 4.1% of lung sections observed, while the therapeutic treatment significantly reduced occupied space to 17.36% ± 4.9% (*p*<0.001). Of interest, there was no change in CFU in the treated group in lung, liver or spleen tissue at four weeks post infection (Fig. S.2), suggesting that (1) the short term administration of lactoferrin did not alter pathogenic burden, and (2) the alteration due to treatments did not result in significant net dissemination to other tissues.

### Increased Penetration of Fluoroquinolone to Inflammatory Foci after Treatment with rHLF

**Fig. 4.**
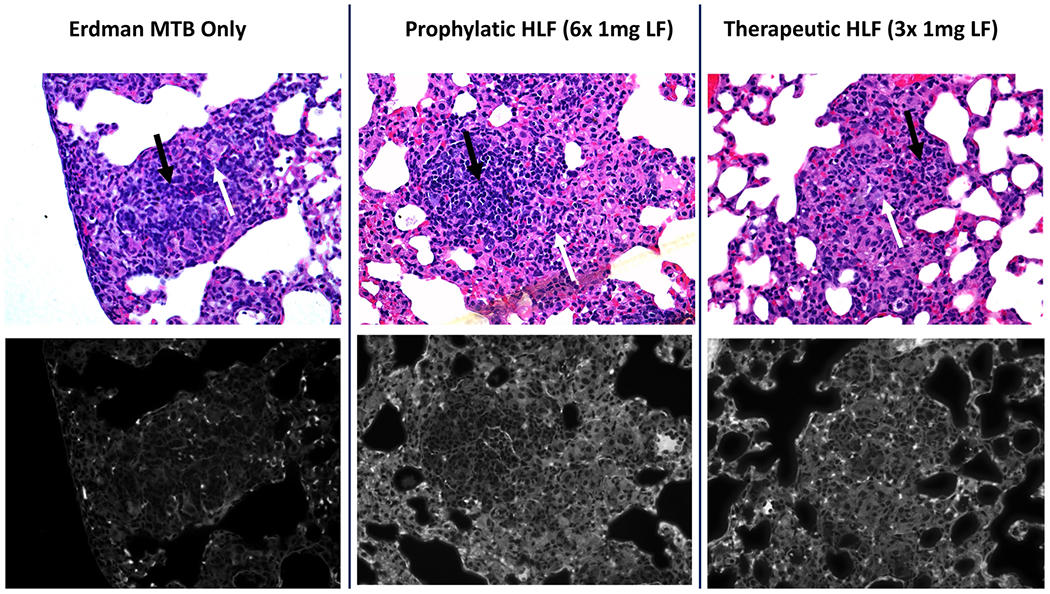
Increased fluoroquinolone penetration to primary granulomas after treatment with lactoferrin. Lungs from *Mtb* infected mice alone (left), or rHLF treated prophylactically (P; middle) or therapeutically (T; right) were assessed for presence of ofloxacin within granulomatous inflammation. Ofloxacin penetration was primarily excluded from inflammatory foci in the *Mtb* infected alone group, while both rHLF treated mice permitted entry of ofloxacin into regions of pathology. Top panels represent hematoxylin & eosin (H&E) brightfield stained histographs (40× magnification) with matching fluorescence captured using multispectral imaging (bottom). Example populations of macrophage-like cells (white arrows) and lymphocytic cells (black arrows) are indicated.

**Fig. 5.**
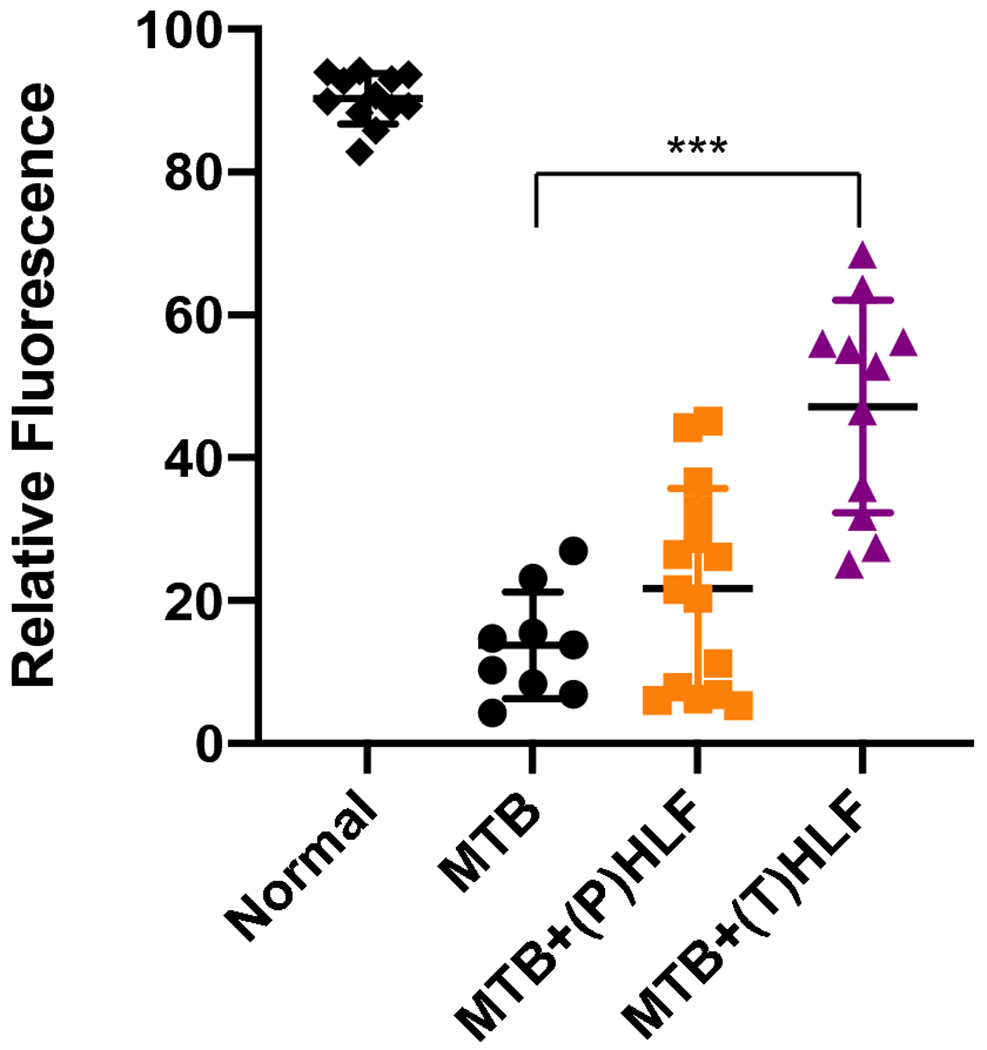
Relative fluorescence for individual inflammatory foci post lactoferrin treatment. Quantitative assessment of ofloxacin penetration into granulomas in lactoferrin treated mice are compared to non-treated infected animals. Individual scans are represented from multiple sections from 6–8 mice per group; average values and standard deviation included. P: prophylactic treatment; T: therapeutic treatment; ***p≤0.001, one way ANOVA, Tukey post hoc test for multiple comparisons.

**Fig. 6.**
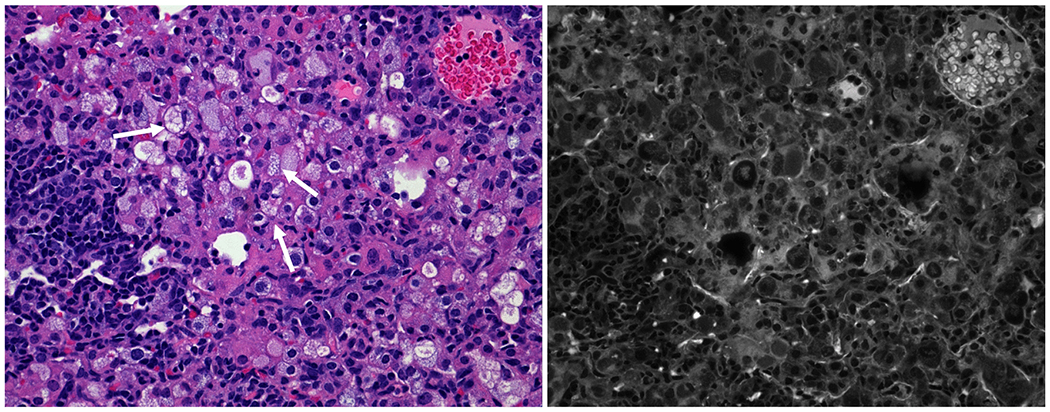
Accumulation of ofloxacin signal in activated macrophages in lactoferrin treated *Mtb* infected mice. H&E staining of inflammatory foci within rHLF therapeutically treated mouse lung revealed acute regions of highly activated “foamy” macrophage-phenotypic cells (white arrows). Mutispectral imaging correlates the presence of fluorescence which overlaps presence of activated cells, located within the focal granulomatous regions. Representative section, 100× magnification.

The altered pathology of less dense granulomatous structures raised the hypothesis that vascular structure would be maintained in the rHLF treated groups, which could subsequently result in enhanced penetration of mycobacterial therapeutic agents to within regions of inflammation. To test this, the naturally fluorescent fluoroquinolone, ofloxacin, was intravenous administered to mice 30 min prior to sacrifice at the 28 days post infection time point. Figure 4 reveals the penetration patterns of ofloxacin to within regions of pulmonary granulomatous response. The histologically dense inflammatory response in the *Mtb* alone group did not permit penetration of the fluoroquinolone, with little to no signal entering granulomatous foci. In contrast, both the prophylactic and the therapeutic rHLF treatment protocols resulted in granulomas that were permissible to ofloxacin penetration. Assessment of serial sections by high resolution scanning revealed significant differences between groups (Fig. 5). The *Mtb* alone infected group had a relative fluorescent distribution of signal 13.76% ± 7.5%, confirming the relative difficulty in penetration of ofloxacin to within inflammatory regions. For comparison, the relative fluorescence of normal mouse lung had an average of 90.25% ± 3.5% penetration, reflecting antibiotic distribution to the lung at 30 min post delivery. Of interest, in alignment with visual observations described above, both rHLF treated groups demonstrated elevated ofloxacin penetration within granulomas. The prophylactic treatment permitted relative increases to 21.68% ± 14.1%, and the therapeutic treatment showed significant increase at 47.15% ± 14.9% presence of fluorescent signal. Furthermore, high power observation of signal revealed accumulation of ofloxacin correlating with presence of activated foamy macrophages (Fig. 6).

### rHLF Treatment Correlates with Retention of Vascular Structure (Endothelial Cell Integrity) in Regions of Pulmonary Inflammation

**Fig. 7.**
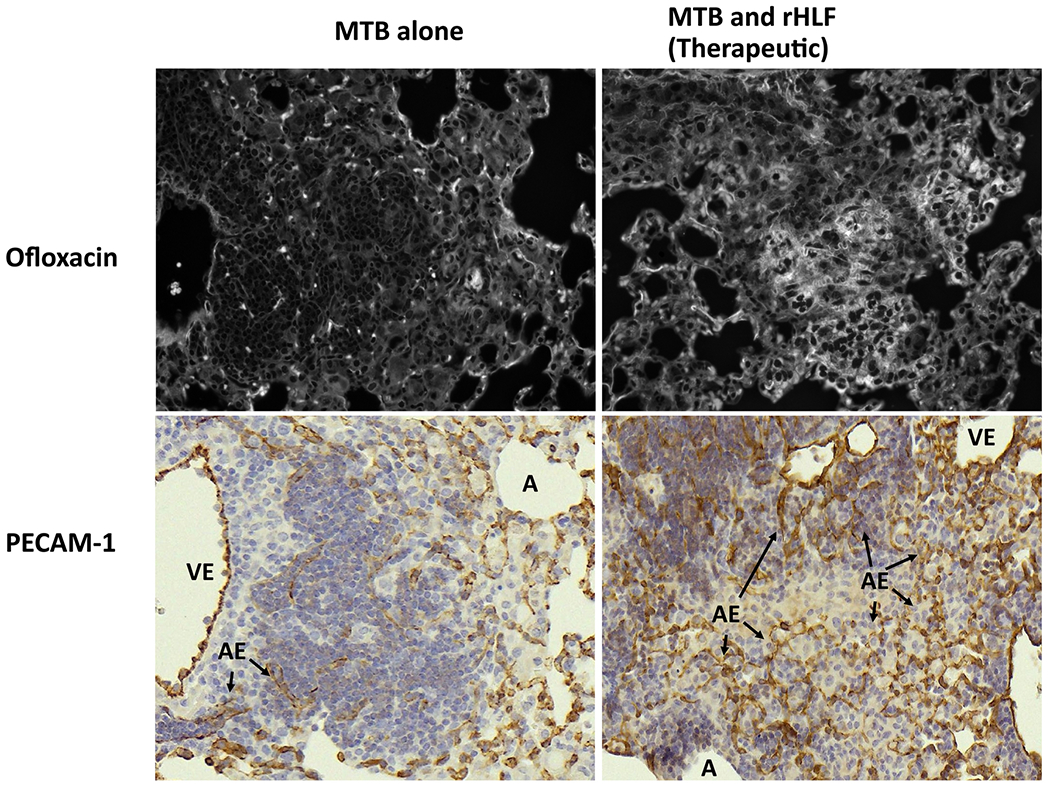
Retention of vascularized structures within regions of inflammation in lactoferrin treated *Mtb*-infected mice. Serial sections compared presence of maintained vascular structure within inflammatory foci in *Mtb* infected mice (left side) or in *Mtb* infected mice treated therapeutically with rHLF (right side). Fluorescence patterns obtained using multispectral imaging (top panels) were compared with serial lung sections that were immunhistochemically stained for PECAM-1 to identify vascular endothelial populations within inflammatory foci (bottom panels). A: alveolus; VE: vascular endothelium; AE: alveolar capillary endothelium (arrows).

The *Mtb* alone infected group demonstrated responses consistent with pulmonary disruption to alveolar structure and associated vascular tissue. To further examine the effect of the rHLF treatment on vascular structure, histological sections were stained with PECAM-1. Granulomas in the *Mtb* alone group demonstrated collapsed alveoli with central accumulation of immune cells in regions that disrupt blood distribution (Fig. 7). Central foci in these mice were devoid of staining, representing reduced vascularization to regions where organisms are expected to reside. In contrast, the rHLF treated mice demonstrated retention of vascularized structures within areas of inflammation, which also corresponded in matched sections to regions demonstrating ofloxacin penetration.

### Altered Localization of M1/M2-Like Macrophages in Primary Granulomas Following rHLF Treatment

**Fig. 8.**
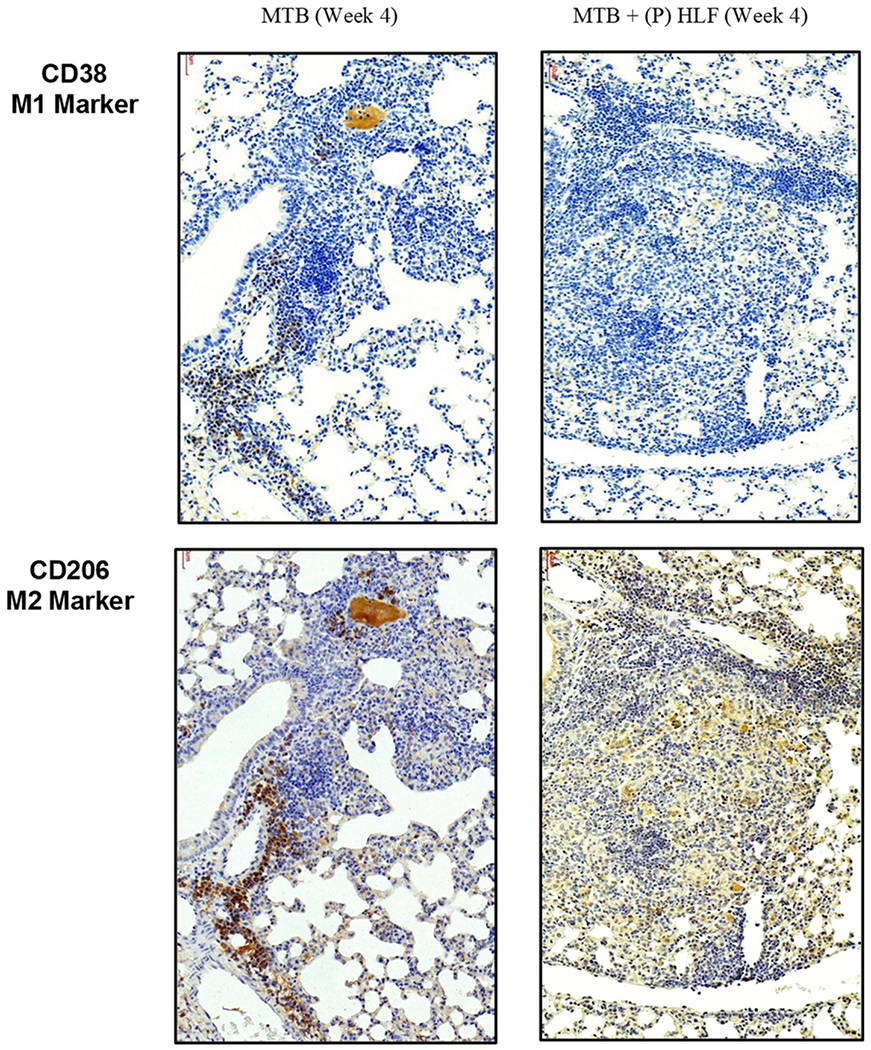
Altered localization of M1-like and M2-like populations following lactoferrin treatment of *Mtb* infected mice. Serial sections of formalin fixed lung tissue were reacted with antibody to CD38 (top) or CD206 (bottom), for either *Mtb* alone (left side) or *Mtb* infected mice treated therapeutically with rHLF (right side). *Mtb* alone infected mice demonstrate accumulation of both M1- and M2-like phenotypic cells in a cuffing pattern surrounding blood vessels adjacent to granulomatous inflammation. A different pattern appears in the rHLF treated group, with minimal presence of M1-like phenotype and a primarily diffuse distribution of M2-like cells throughout the inflammatory foci.

Recent reports demonstrated altered distribution of macrophage populations within primary granulomas, which may dictate pathological outcomes (Pisu et al. 2020a, 2020b). As a preliminary investigation into this topic, sections were immunohistochemically stained for general M1-like and M2-like antigens. Figure 8 depicts immunohistochemical staining for the M1-like marker CD38 and for the M2-like marker CD206. The non-treated *Mtb* infected lungs revealed a concentrated pattern of staining demonstrating high presence of both M1-like and M2-like macrophages, primarily cuffing vascular regions surrounding regions of inflammation. Limited numbers of both the M1-like and the M2-like cells were visible within the granuloma itself in the *Mtb* alone infected group. In contrast, the rHLF treated mice exhibited a only small number of M1-like cells that were lightly stained, residing inside the granulomatous response. Of note, the M2-like staining pattern in the lactoferrin treated groups showed M2-like macrophages which were evenly and diffusely distributed throughout the primary granulomatous pathology.

## DISCUSSION

This is the first report that recombinant human lactoferrin can modulate the early granulomatous response during primary *Mtb* infection in mice, in a manner nearly identical to that reported for bovine lactoferrin (Welsh et al. 2011). The results presented indicate utility for human lactoferrin administered orally as a therapeutic approach to limit pathological damage during primary granuloma development. The intervention led to increased penetration of ofloxacin to regions where *Mtb* typically reside. Our observations in this model are in line with studies examining induced granulomas using purified mycobacterial mycolic acid TDM (Nguyen et al. 2021), where administration of rHLF achieved inflammation reduction in the lungs and greater ofloxacin distribution throughout granulomatous structures after treatment.

Lactoferrin-induced modulation in inflammatory response within mycobacterial-induced granulomas is likely the result of two concurrent events; that of significantly less proinflammatory cytokine production and reduction in recruited M1-like macrophages (Nguyen et al. 2021). Alveolar macrophages uptake *Mtb* during primary infection and become essentially the key cell phenotype to recruit and activate additional naïve macrophages to sites of infection (Akira et al. 2006; McClean and Tobin 2016). Classically activated macrophages polarize to the M1-like phenotype and exhibit functional phagocytosis and killing of bacteria. As a pathological by-product, they initiate immune cell recruitment via secreted proinflammatory cytokines to aid in the establishment of granulomatous tissue structures. Temporally, the subsequent recruitment and introduction of M2-like macrophages allows immune modification of the aggressive inflammatory response, permitting an immunological “brake” to an overwhelming pathological response (Hunter et al. 2006a; Huang et al. 2015; Marino et al. 2015; Nguyen et al. 2020; Thiriot et al. 2020). While further investigation is required, it is theorized that a balance in the presence of macrophage phenotypes within the granulomatous structures is essential to control pathological mediators by innate immune cells (Pisu et al. 2020a, 2020b), which is critical to regulation of IL-1β (T cell activation and migration) (Schmitz et al. 2005) and TNF-*α* (vasodilatation and leukocyte adhesion to epithelium) (Page et al. 2018) necessary as a response to control bacterial growth.

A major observation of this study was that the second-line mycobacterial therapeutic fluorquinolone was able to penetrate within inflammatory foci. Similar to reported results using the TDM non-infectious model of pathology, significant amount of ofloxacin was found within granulomas following treatment with human lactoferrin, especially, but not limited to, regions of reduced inflammation. Concurrently, the lactoferrin-treated mice exhibited granulomatous responses with maintained vascular structures and open alveolar spaces. Acute inflammation and reactivity during *Mtb* infection occurs primarily peripheral to vascular regions, coinciding with destruction in continuity of endothelial lined blood vessels (Hunter et al. 2018; Hwang et al. 2019). In the experiments presented in this report, lactoferrin treatment allowed greater regions of lung tissue, and alveolar spaces, to remain unobstructed, as evident using the PECAM-1 endothelial surface marker. This coincided with observational retention of blood vessels within regions of pathology. The lessened pathological damage in the lactoferrin treated animals, along with significant maintenance of vascular architecture, likely permitted ofloxacin transport inside of granulomas, as evident by the drug’s fluorescent signal observed within and around the endothelial-lined vessels. Therefore, we hypothesize the promising use of lactoferrin as a safe adjuvant molecule to increase delivery of standard *Mtb* therapeutics, especially in newly exposed cohorts of post-primary status individuals. This may also have the potential to reduce overall treatment times, limit drug sensitivity development, and reduce antibiotic side effects in patients undergoing treatment. At this time, it is certainly recognized that the line of research using fluoroquinolone may not extend to other classes of antimycobacterial agents.

A major concern with current host-directed therapy is the increase of bacterial dissemination during treatment, such as seen when TNF-α blockers are used (Chakravarty et al. 2008; Fernandez-Ruiz and Aguado 2018; Keane et al. 2001; Wallis et al. 2004). It is crucial that the role of established granulomas is maintained throughout the anti-mycobacterial treatment; specifically, the activation of recruited immune cells must be maintained to limit organism spread to other tissues. Our observations (supplemental data) demonstrated no increase in lung CFUs in the treated group and no change in levels of detected organisms in liver and spleen. Such observation aligns with previous studies using bovine lactoferrin in a similar *Mtb* infectious model (Welsh et al. 2011). This shows potential therapeutic application of recombinant human lactoferrin to reduce pathological damage due to infection. And it does so without the major side effect of other host-directed therapies, including the compromising of *Mtb* confinement at the site of initial inflammation.

Lactoferrin immune-modulating effects in our *Mtb* infectious model are consistent with other infectious models reported (Actor et al. 2009; Drago-Serrano et al. 2017; Sienkiewicz et al. 2021). Its utility as a host-directed therapeutic has been demonstrated in other diseases where host inflammation plays a key role in pathology development (Actor et al. 2009; Doursout et al. 2013). Lactoferrin was used successfully in clinical trials a prophylactic to prevent development of enterocolitis and sepsis (Manzoni et al. 2009; Ochoa et al. 2012; Turin et al. 2014). In a similar manner, the prophylactic administration presented here was done prior to the establishment of granulomatous pathology. However, significant results were surprisingly identified when lactoferrin was given as a therapeutic at day 21 post infection at a time after granulomas have initiated in the lungs. Of clinical importance, when lactoferrin was used therapeutically it both decreased overall lung inflammation and increased ofloxacin distribution in granulomas.

Mechanistically, lactoferrin can induce dendritic cell activation and maturation, specifically supporting functions of antigen-presenting cell populations that act as to bridge innate and adaptive immunity (de la Rosa et al. 2008; Hwang and Actor 2009; Hwang et al. 2015; Rascon-Cruz et al. 2021; Spadaro et al. 2014). Lactoferrin can directly enhance the antigen presenting activity and T cell stimulation function in mycobacterial-infected macrophages (Hwang et al. 2009b). Lactoferrin can also modulate T cell activities in multiple ways (Actor et al. 2009). *In vitro*, T cell maturation and expression of T cell ζ-chain, a component of T cell receptor complex involved in receptor signaling pathways, can be increased upon treatment with lactoferrin (Dhennin-Duthille et al. 2000; Frydecka et al. 2002; Zimecki et al. 1991); T cell adhesive molecules involved in cell-to-cell contact are also increased in the presence of lactoferrin (Zimecki et al. 1999). Clearly, lactoferrin can influence T helper cell polarization (Fischer et al. 2006). Bovine lactoferrin administered orally functions to induce both systemic and mucosal responses (Debbabi et al. 1998), with specific increases in IFN-γ Th1 T cell responses (Takakura et al. 2006) and NK cell activity in mice (Kuhara et al. 2006) likely by increasing IL-12 and related cytokines (Hwang et al. 2007; Kuhara et al. 2006). Together, these suggest possible modulations of T cell activities, combined with enhanced antigen-presenting cell maturation and macrophage recruitment effects, which theoretically shift the overall outcome of granulomas to successful containment of pathogens

Previous observations using the TDM-induced granuloma model demonstrated that lactoferrin could lessen the presence of inflammatory mediators post initiation of pathology (Nguyen et al. 2021), as well as preferentially recruit M2-like cells to granulomas (Nguyen et al. 2021). It is reasonable to infer that similar reductions using human lactoferrin at four weeks post *Mtb* infection would occur, since TDM is significantly released from mycobacteria (Hunter et al. 2006a, 2006b). This also suggests an important mechanistic link between macrophage recruitment and lactoferrin treatment during *Mtb* infection, perhaps via the alteration of surface adhesion markers on monocytes that would permit increased interaction with endothelial cells (Baveye et al. 2000; Yeom et al. 2011). However, it remains unknown if lactoferrin can differentially affect M1-like versus M2-like activities; such experiments have only been explored on classical M1-like activated monocytes (Hwang et al. 2009b; Nguyen et al. 2020). This suggests a premise that macrophage polarization could occur in the presence of lactoferrin, perhaps via the reduction of locally produced proinflammatory cytokines (Kruzel et al. 2002; Mantovani et al. 2004; Nguyen et al. 2021; Thiriot et al. 2020; Wang et al. 2014). And it is unknown at this time if mouse lactoferrin present in the lungs would act in a synergistic manner with the endogenously delivered molecule.

The studies presented here set a foundation to further investigate usage of human lactoferrin in combination with standard treatments for *Mtb* infection. Bacterial dissemination in later time points after infection, and prolonged treatments, should be studied to understand the full potential of its use as a host-directed therapeutic. In reality, it is not clinically feasible to identify individuals prior to development of pathology and granuloma formation. Therefore, more experiments are needed to determine the extent of human lactoferrin on the adaptive immune response when treatments begin after exposure at the primary granuloma establishment and maintenance stages. Furthermore, these studies were done with a focus on the early granulomatous response; experiments should include effects on caseous granulomatous, such as that seen when using the pathologically aggressive C3HeB/FeJ mouse model (Driver et al. 2012). Additional avenues of research should include lactoferrin delivery routes (Sfeir et al. 2004); it may be advantageous for directed aerosol delivery to regions most affected during tuberculosis infection. Overall, a role for clinical utility of human lactoferrin to modify the aggressive immune function during primary *Mtb* infection may exist, which would allow greater efficacy of treatments. In turn, this would potentially reduce standard treatment duration, antibiotic side effects, and overall pathological damage in patients.

## Supplementary Material

1785812_Sup_material **Fig. S.1 Lactoferrin treatment reduces pulmonary inflammation post infectious challenge with *Mtb* in a dose dependent response**. Lungs from *Mtb* infected mice were assessed at day 28 post aerosol infection (A) and compared to animals given bovine LF in the prophylactic group (B, C) or therapeutic group (D, E). Histologic assessment revealed primary granulomatous response with monocytic cell infiltration, dense cellular foci, and occluded vascular regions in control infected mice. Both prophylactic and therapeutic rHLF treatment reduced inflammatory response resulting in modest inflammatory foci and reduced pathological damage to lung tissue. While both doses (100 μg and 1 mg levels) were productive in limiting focal inflammation, the higher dose was more consistent between treatment groups. Hematoxylin and eosin stained histographs represent formalin fixed lung sections at 10× magnification obtained with 8–10 mice in each group; study representative of repeat experiments.
**Fig. S.2. Mycobacterial burden in lactoferrin treated mice.** C57Bl/6 mice were aerosol challenged with *Mtb*, strain Erdman, and treated with bovine lactoferrin (bLF) given as 100 μg or as 1 mg dose administered every other day orally beginning on day 14 (prophylactic treatment), or beginning on day 21 (therapeutic treatment) post infection. Lung (A), spleen (B) and liver (C) were removed on day 28 post infection; tissues were assessed for bacterial CFUs confirmed by plating serial dilutions on Middlebrook 7H11 agar plates using the large right lobe of the mouse lung that was weighed and homogenized into 2 mL PBS, which were subsequently incubated at 37°C for 3–4 weeks and represented as CFU burden per organ. Data are presented as individual mice with the mean and SEM indicated, *n* ≥ 6 mice per group.
**Fig. S.3. CellProfiler analysis of ofloxacin in histological sections.** Example analysis is presented for pulmonary sections taken at 8 weeks post infection with MTB alone (A) or with lactoferrin treatment beginning at day 14 through day 28 (B). Each scanned section was assessed according to threshold parameters indicated. Counter-clockwise from top left shows (1) 100× histological scanned image; (2) single color outline of penetrating fluorescence; (3), color coding of ofloxacin signal detected (minus background), and (4) CellProfiler program threshold parameters Program parameters assume multiple objects in the image; colors indicate non-continuous regions of fluorescence highlighting separation of detected fluor to regions of intracellular accumulation.
