# Supplementary material for: Recombinant Human Lactoferrin Reduces Inflammation and Increases Fluoroquinolone Penetration to Primary Granulomas during Mycobacterial Infection of C57Bl/6 Mice
Source: Arch Immunol Ther Exp (Warsz). Author manuscript; Available in PMC 2023 Feb 28. (PMC8922470; doi:10.1007/s00005-022-00648-7)
Supplement: 1785812_Sup_material — Fig. S.1 Lactoferrin treatment reduces pulmonary inflammation post infectious challenge with Mtb in a dose dependent response. Lungs from Mtb infected mice were assessed at day 28 post aerosol infection (A) and compared to animals given bovine LF in the prophylactic group (B, C) or therapeutic group (D, E). Histologic assessment revealed primary granulomatous response with monocytic cell infiltration, dense cellular foci, and occluded vascular regions in control infected mice. Both prophylactic and therapeutic rHLF treatment reduced inflammatory response resulting in modest inflammatory foci and reduced pathological damage to lung tissue. While both doses (100 μg and 1 mg levels) were productive in limiting focal inflammation, the higher dose was more consistent between treatment groups. Hematoxylin and eosin stained histographs represent formalin fixed lung sections at 10× magnification obtained with 8–10 mice in each group; study representative of repeat experiments. Fig. S.2. Mycobacterial burden in lactoferrin treated mice. C57Bl/6 mice were aerosol challenged with Mtb, strain Erdman, and treated with bovine lactoferrin (bLF) given as 100 μg or as 1 mg dose administered every other day orally beginning on day 14 (prophylactic treatment), or beginning on day 21 (therapeutic treatment) post infection. Lung (A), spleen (B) and liver (C) were removed on day 28 post infection; tissues were assessed for bacterial CFUs confirmed by plating serial dilutions on Middlebrook 7H11 agar plates using the large right lobe of the mouse lung that was weighed and homogenized into 2 mL PBS, which were subsequently incubated at 37°C for 3–4 weeks and represented as CFU burden per organ. Data are presented as individual mice with the mean and SEM indicated, n ≥ 6 mice per group. Fig. S.3. CellProfiler analysis of ofloxacin in histological sections. Example analysis is presented for pulmonary sections taken at 8 weeks post infection with MTB alone (A) or with lactoferrin treatment be [file NIHMS1785812-supplement-1785812_Sup_material.pptx]

## Slide 1
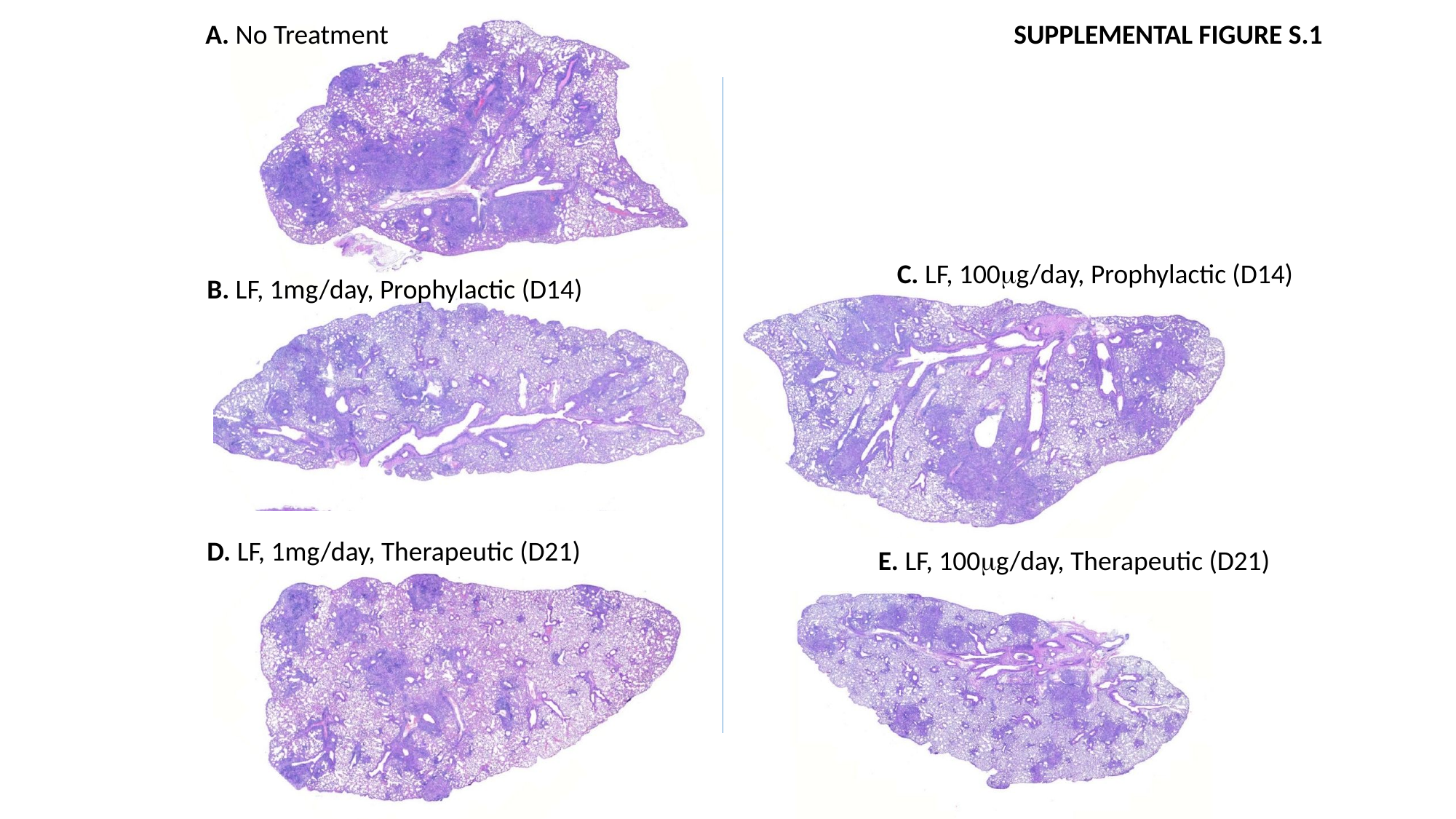

A. No Treatment
SUPPLEMENTAL FIGURE S.1
C. LF, 100μg/day, Prophylactic (D14)
B. LF, 1mg/day, Prophylactic (D14)
D. LF, 1mg/day, Therapeutic (D21)
E. LF, 100μg/day, Therapeutic (D21)

## Slide 2
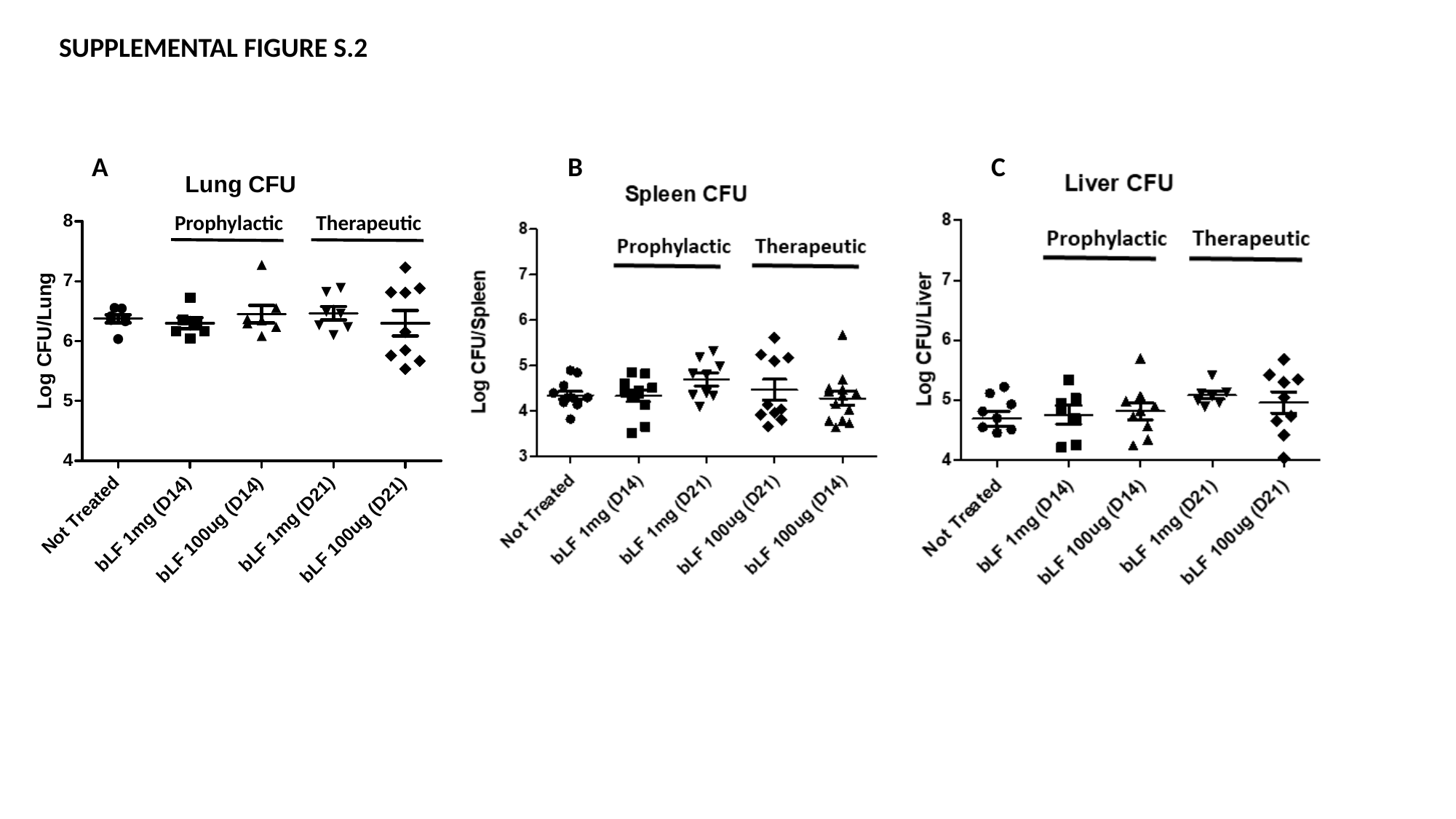

SUPPLEMENTAL FIGURE S.2
A
B
C
Prophylactic Therapeutic

## Slide 3
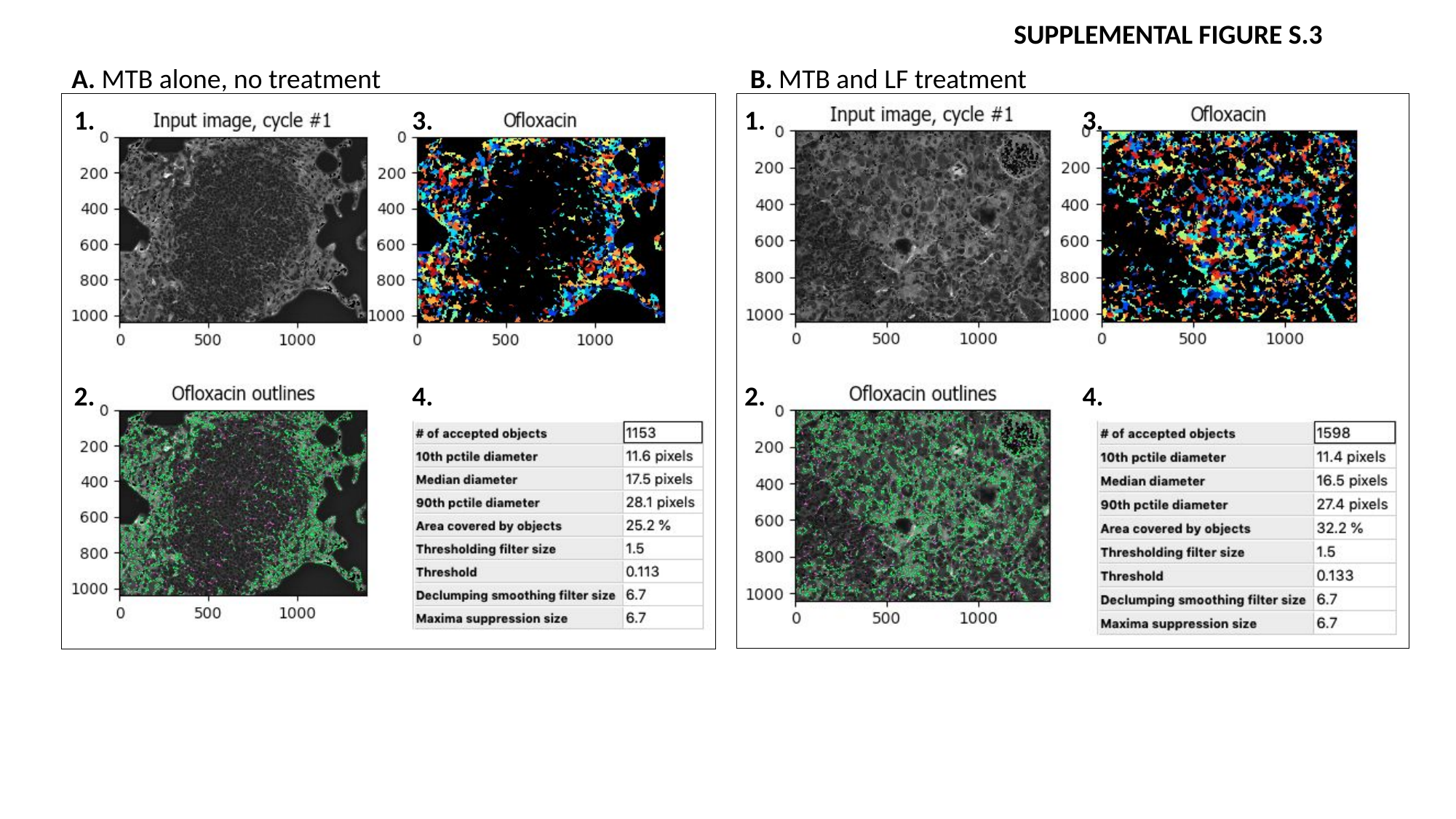

SUPPLEMENTAL FIGURE S.3
A. MTB alone, no treatment
B. MTB and LF treatment
1.
3.
2.
4.
1.
3.
2.
4.
